# Efficient Conversion of Agroindustrial Waste into D(-) Lactic Acid by *Lactobacillus delbrueckii* Using Fed-Batch Fermentation

**DOI:** 10.1155/2020/4194052

**Published:** 2020-04-22

**Authors:** Susan Michelz Beitel, Luciana Fontes Coelho, Jonas Contiero

**Affiliations:** ^1^Department of Biochemistry and Microbiology, Institute Bioscience, São Paulo State University (UNESP), Av. 24A 1515 CEP- 13506-900, Rio Claro São Paulo, Brazil; ^2^Associate Laboratory IPBEN-UNESP, Av. 24A 1515 CEP- 13506-900, Rio Claro São Paulo, Brazil

## Abstract

**Purpose:**

The goal of this paper is to describe the green conversion of agricultural waste products, such as molasses and corn steep liquor, into large amounts of D(-) lactic acid using a facilitated multipulse fed-batch strategy and affordable pH neutralizer. This is a very low-cost process because there is no need for hydrolysis of the waste products. The fed-batch strategy increases lactic acid productivity by avoiding inhibition caused by a high initial substrate concentration, and the selected controlling agent prevents cell stress that could be caused by high osmotic pressure of the culture media.

**Methods:**

The effects of different carbon and nitrogen sources on lactic acid production were investigated, and the best concentrations of the medium components were determined. To optimize the culture conditions of the *Lactobacillus delbrueckii* strain, the effects of pH control, temperature, neutralizing agent, agitation, and inoculum size in batch cultures were investigated. Fed-batch strategies were also studied to improve production and productivity.

**Result:**

A high titer of D(-) lactic acid (162g/liter) was achieved after 48 hours of fermentation. Productivity at this point was 3.37 g/L·h. The optimum conditions were a temperature of 39°C, pH 5.5 controlled by the addition of Ca(OH)_2_, agitation at 150 rpm, and inoculum size of 25% (*v*/*v*).

**Conclusion:**

The production of high optical purity D(-) lactic acid through *L*. *delbrueckii* fermentation with molasses and corn steep liquor is a promising economical alternative process that can be performed on the industrial scale.

## 1. Introduction

Lactic acid has gained considerable attention due to its role as a monomer for the production of biodegradable polylactic acid (PLA). It is also widely used in the food, cosmetic, pharmaceutical, leather, textile, and chemical industries [1–4]. Several methods have been studied in an attempt to improve lactic acid fermentation with the goal of achieving higher yields [5].

Lactic acid has two optical isomers: L(+) and D(-). Optically pure L(+) or D(-) lactic acid can be achieved through microbial fermentation using an appropriate microorganism that is capable of producing one of these isomers. In contrast, chemical synthesis always leads to the production of racemic DL-lactic acid [6, 7]. The optical purity of lactic acid is an important physical property that has attracted the attention of different industries that blend L(+) or D(-) polymers to polymerize high-crystalline PLA, thereby improving the mechanical properties of this polymer [8, 9].

There are numerous investigations on the development of biotechnological processes for the production of L(+) lactic acid and industrial-scale production has been established [10–14]. However, fewer studies have investigated D(-) lactic acid production by fermentation processes. On the industrial scale, lactic acid is generally produced using batch fermentation, but this method has the disadvantage of decreasing production and productivity due to inhibition by the high substrate concentration [15, 16]. Thus, different fed-batch fermentation strategies have been explored to enhance productivity and avoid the inhibitory effects of the substrate on lactic acid production [17–19].

Several bacterial species belonging to the genus *Lactobacillus*, such as *plantarum*, *delbrueckii*, *sakei*, *casei*, and *lactis*, have been widely used for the production of lactic acid [19–23]. The production cost and demand of lactic acid to make it an environmentally feasible process are important aspects driving the search for improvements in this technology [24]. Studies report the production of lactic acid using raw materials as substrate, such as rice starch, barley, wheat, corn, cottonseed meal, potato peel waste, fruit, and vegetable waste [17, 25–29]. However, these sources usually require pretreatment in order to release fermentable sugars, which is economically unfavorable [30]. The use of waste products from industrial processes, such as molasses, which contains fermentable sugars, and corn steep liquor, is a very economical alternative, and these waste products can be readily used as nutritional sources in fermentation media, thereby decreasing the overall production cost [22, 30, 31].

Molasses is a byproduct of the sugar industry that contains 40 to 60% sucrose, which can be converted into lactic acid through fermentation [6, 30]. Molasses is used mainly as a carbon source and requires supplementation with nitrogen and minerals in order to be used in fermentation media [32]. Corn steep liquor is a waste product generated by the corn industry that contains amino acids, vitamins, and minerals, which makes it an excellent nitrogen source that can used by microorganisms [33].

Corn is currently the most widely produced cereal in the world, with an estimated 989.30 million tons produced in the 2015/2016 season. Brazil is among the three largest producers of corn, along with China and the USA [34]. Brazil is also the largest sugarcane producer and the world leader in sugar production. In this process, large amounts of molasses are generated as a byproduct produced at the proportion of 40 to 60 kilograms per ton of processed sugarcane [35, 36]. In an attempt to establish cost-effective technologies, this paper describes the efficient conversion of agroindustrial waste products (molasses and corn steep liquor) into lactic acid by *L. delbrueckii* using fed-batch fermentation.

## 2. Material and Methods

### 2.1. Microorganisms and Seed Cultivation Conditions

*L*. *delbrueckii* used in the present study was isolated from commercial yogurt and stored using a previously reported technique [37]. The seed medium was cultured in GYP medium (glucose (2%), yeast extract (1%), peptone (1%), sodium acetate (1%), and 0.5% of salt solution that comprises MgSO_4_.7 H_2_O (4%), MnSO_4_. (0.2%), FeSO_4_. 7H_2_O (0.2%), and NaCl (0.2%); all of these reagents are of Analytical Purity-Synth) for 15 h at 35°C under stationary conditions until OD_600_ reached 5.0 and then inoculated with 10% (*v*/*v*) in Erlenmeyer flasks or the fermentor (except in the test on the influence of inoculum size on lactic acid production).

### 2.2. Optimization of Fermentation Medium

#### 2.2.1. Influence of Carbon and Nitrogen Sources on Lactic Acid Fermentation

The effect of different carbon sources on lactic acid production by *L*. *delbrueckii* was studied using molasses, commercial sucrose, and whey as the substrate at a concentration of 140 g/L of sugar content based in the GYP medium. After selecting the carbon source, different nitrogen sources were evaluated, such as corn steep liquor, Proflo, yeast autolysate, peanut flour, soybean flour, ammonium sulfate, urea, and ammonium nitrate. The concentration of the nitrogen sources was based on the GYP medium. Fermentation was carried in 125 mL flasks containing 50 mL of the medium at pH 7.0 and 10% inoculum (*v*/*v*) under stationary conditions at 35°C with incubation for 48 hours. To avoid the decrease in pH due to the production of lactic acid, 5% CaCO_3_ was added to each flask before sterilization. The experiments were run in duplicate, and the results were expressed as mean values.

#### 2.2.2. Response Surface Methodology (RSM)

The concentrations of the carbon source, nitrogen source, and mineral salts were studied using RSM, which is a set of experimental strategies applied to determine the best concentration of medium components that exert an influence on D(-) lactic acid production. For such, 17 experiments were run, including three replicates at the center point. The independent variables selected for the study were the concentration of molasses, corn steep liquor, and mineral salts (the same used in the GYP medium).

The dependent variable (D(-) lactic acid production) was fitted using a second-order equation, and response surface graphs were then created. Lactic acid fermentation was performed under the same conditions listed in the previous topic. The experimental design was determined using the Statistica 7 software program (StatSoft, Tulsa, USA), which was also used for the analysis of the results.

#### 2.2.3. Study of D(-) Lactic Acid Fermentation Conditions

Fermentations were performed in a 750 mL bioreactor (Infors HT Multifors 2) with an initial working volume of 300 mL of optimized medium containing 150 g/L of molasses (except for the agitation and inoculum size experiments, which began with 160 g/L), 65 mL/L of corn steep liquor, and 5 mL/L of mineral salts. The system was equipped with pH and temperature sensors as well as feed and base pumps. The effects of the pH-controlling agent (Ca(OH)_2_, CaCO_3_, NaOH, NH_4_OH, and KOH), temperature (31°, 33°, 35°, 37°, 39°, and 41°), pH (5.0, 5.5, 6.0, 6.5, and 7.0), agitation (50, 75, 100, 125, and 150 rpm), and inoculum size (5, 10, 15, 20 25, and 30%) on lactic acid production were investigated.

The results are described below. The fermentation products were sampled periodically and analyzed to determine the concentrations of the substrate and lactic acid.

#### 2.2.4. Neutralizing Agents

Lactic acid produced through fermentation has to be continuously neutralized, and the pH-controlling agent exerts an influence on lactic acid production. The effect of acid neutralization on fermentation performance was investigated using different pH-controlling agents: Ca(OH)_2_ 9N, NaOH 10N, NH_4_OH 6N, CaCO_3_ 5%, and KOH 10N. The reactors were mechanically stirred at 100 rpm and maintained at 35°C. The pH was maintained at 6.0 by the automatic addition of each neutralizing agent. The medium (270 mL) was inoculated with 30 mL of standardized culture.

#### 2.2.5. Effects of Fermentation Parameters on Production of D(-) Lactic Acid

A set of experiments was conducted to evaluate the effects of temperature, pH, agitation, and inoculum size on lactic acid fermentation. The cultivation parameters used in these experiments are given in Table 1. Fermentations were carried out for 70 hours, and Ca(OH)_2_ 9N was used as the pH-controlling agent.

#### 2.2.6. Fed-Batch Fermentations

Fed-batch fermentation is used to prevent inhibition due to a high initial substrate concentration. For such, nutrients are supplied either intermittently or continuously to the bioreactor throughout the cultivation process [38, 39].

In the present study, different fed-batch fermentations of *L*. *delbrueckii* were implemented using pulse, multipulse, and continuous feeding rate strategies. The fermentations were carried out in a 750 mL bioreactor (Infors HT Multifors 2) with an initial medium containing 65 mL/L of corn steep liquor, 5 mL/L of mineral salt solutions (the same as the GYP medium), and different concentrations of molasses, as described below. The processes were run under the previously optimized conditions at 39°C, 150 rpm, and pH 5.5 controlled by the automatic addition of Ca(OH)_2_ 9N. The initial working volume was 300 mL with 25% inoculum (*v*/*v*). The feed solution was composed of 585 g/L of sucrose from molasses and 65 mL/L of corn steep liquor and was pumped into the bioreactor by a computer-controlled peristaltic pump as required. For the comparison of the processes, batch fermentation was performed under the same culture conditions containing 160 g/L of initial sucrose from molasses, with the addition of only the pH-controlling agent during the process.

In the pulse fed-batch process, the initial molasses concentration was set to 100 g/L and after 15 hours; when no residual sucrose was present, a single pulse was applied into the bioreactor with the addition of 60 g/L of sucrose in the feed solution. Another single-pulse fermentation process was carried out in which the initial concentration of molasses was 150 g/L, and 55 g/L of sucrose were added when the residual sugar was near 25 g/L. The multipulse fed-batch process began with 100 g/L of molasses, and the feed solution was supplied twice, when the residual sugar was below 10 g/L; 45 g/L and 30 g/L of sucrose from the feed solution were added at 15 and 24 hours, respectively. In constant feed rate fed-batch fermentation, when all the initial sucrose was consumed (at 15 hours), the feed solution was continuously pumped into the bioreactor at a rate of 7.58 mL/h until completing 24 hours of fermentation. Fermentations were conducted for 70 hours. Samples were collected periodically to determine the concentrations of lactic acid and sugar as well as cell growth.

#### 2.2.7. Analysis of Sugars, Lactic Acid and Cell Growth

The samples collected were centrifuged at 7000xg for 15 minutes, microfiltered through a 0.22 *μ*m membrane, and the supernatants were used to determine the concentration of residual sugar and D(-) lactic acid. These concentrations and the optical purity of the lactic acid were determined using high-performance liquid chromatography (HPLC), as reported elsewhere [37].

The cell concentration was determined using a calibration curve (*y* = 1.1297*x* − 0.045) correlating the optical density at 600 nm (measured in the spectrophotometer) to the dry cell weight, which was determined by centrifugation at 7000xg for 15 minutes, washing twice (first with HCl 0.3 N and then with distilled water), followed by drying at 100°C for 24 hours. The sugar from whey is lactose that induced the lower concentration of lactic acid on the present study; on the other hand, it was reported that the highest yield of D(-) lactic acid from lactose substrate derived from whey.

## 3. Results and Discussion

### 3.1. Effects of Carbon and Nitrogen Sources on D(-) Lactic Acid Production

*L*. *delbrueckii* was able to produce D(-) lactic acid using all the carbon sources studied; however, the highest production (112.84 ± 3.07 g/L) was achieved in the presence of molasses, with 12.12 ± 1.22 g/L of residual sucrose. Commercial sugar led to a similar lactic acid concentration (111.37 ± 1.15 g/L) but higher residual sucrose value (24.98 ± 1.22), as displayed in Table 2. The sugar from whey is lactose, which in this study induces the lower D(-) lactic acid production (100.08 ± 2.06). On the other hand, efficient conversion of this sugar into lactic acid by another strain of *L. delbrueckii* has been reported [40–42]. Another author reported that among various carbon sources including glucose, sucrose, molasses, sugarcane juice, and bagasse hydrolysate, greater lactic acid production was observed using sucrose as the carbon source [43].

Molasses may provide greater cell growth due to the presence of nitrogen in its composition as well as its buffering action, as it contains calcium [30]. Molasses is a byproduct of the sugar industry. It is a syrupy material left after the removal of sugar from the mother syrup and is available in large amounts in Brazil, which is the largest sugarcane grower and sugar producer on the global scale, accounting for more than half of the sugar sold worldwide. Molasses offers a considerable advantage over current readily fermentable sources of sucrose, as it does not require pretreatment or hydrolysis [36, 44, 45]. Previous studies have reported the conversion of molasses to lactic acid by *L*. *delbrueckii* [44, 45]. However, some researchers submitted the molasses to hydrolysis with H_2_SO_4_ [30–46]. In the present study, *L*. *delbrueckii* was able to metabolize molasses without hydrolysis, which makes the process more economical.

Among the nitrogen sources studied, yeast autolysate and corn steep liquor led to the highest lactic acid production and productivity, performing similarly. However, corn steep liquor is a byproduct of the corn industry and is therefore a very inexpensive material. The use of agroindustrial waste products in fermentation processes is an environmentally friendly approach that enables the conversion of waste into added-value products [47]. Lactic acid production was not favored by ammonium nitrate, ammonium sulfate, or urea, as these substances are inorganic nitrogen sources, and considering that the base medium does not contain any type of vitamin, which is important to the maintenance of microbial cells. Assavasirijinda et al. [9] report similar results in an investigation of D(-) lactic acid production by an engineered *Bacillus* sp. strain, in which inorganic nitrogen sources did not lead to satisfactory levels of production, whereas the highest lactate production was achieved using peanut meal as the nitrogen source. In the present study, higher lactic acid concentrations were found when using waste products (corn steep liquor and Proflo), which have vitamins in their compositions, such as thiamine, riboflavin, niacin, pantothenic acid, choline, pyridoxine, biotin, inositol, carotene, tocopherols, ascorbic acid, and folic acid. Thus, molasses and corn steep liquor can be considered alternative substrates for lactic acid fermentation, as these products satisfy the nutrient requirements of *L*. *delbrueckii*.

### 3.2. Optimization of Fermentation Medium Using Response Surface Methodology

The mathematical relationship for D(-) lactic acid production was developed by considering three independent variables (concentrations of molasses, corn steep liquor, and mineral salt solution) and one dependent variable (D(-) lactic acid concentration in final medium fermentation). The response values of productivity, yield, and residual sucrose for each run are also shown (Table 3).

At the end of the fermentation process, the highest titer of lactic acid (124.56 g/L), highest productivity (2.60 g/L·h), and residual sugar of 36.30 g/L occurred in run 15. Central point values were used in this assay.  Table 4 displays the results of the analysis of variance for the response surface quadratic model.

The regression equation had an *R*2 value of 0.94, which demonstrates the satisfactory fit of the quadratic model to the experimental data and indicates that the model explains 94% of the variability in the response. The model was significant, as demonstrated by Fisher's *F* calc test (13.08) > Ft (3.39), and had a very low probability value (Pmodel = 0.001). Moreover, the lack-of-fit value was nonsignificant at the 5% level (*p* > 0.05), indicating the satisfactory predictability of the model. A probability (*p*) value < 0.05 is indicative of a statistically significant coefficient.

Interactions *X*1*X*1, *X*2*X*2, and *X*3*X*3 were significant with 95% probability, demonstrating a negative effect. The high sucrose content of molasses can induce catabolic repression. Moreover, high mineral salt concentrations affect the membrane permeability of bacterial cells as well as the osmotic pressure of the medium, which may lead to cell lysis or hinder the passage of essential nutrients through the microbial cell membrane. Corn steep liquor contains minerals in its composition (calcium (0.14%), copper (1.5 mg/100 g), manganese (2.0 mg/100 g), iron (10 mg/100 g), magnesium (0.6%), potassium (2.8%), sodium (0.1%), and phosphorus (1.8%)) as well as impurities [48], which, when combined with the mineral salt solution used in this experiment, results in a high salt concentration and may have caused the effects abovementioned in the cells of *L*. *delbrueckii*. The results achieved were submitted to multiple linear regression analysis to generate the following regression equation (1):

(1) $$\begin{array}{c} Y=122.24+0.25X_{1}-18.67X_{1}X_{1}+2.07X_{2}-11.37X_{2}X_{2}+2.15X_{3}-6.59X_{3}X_{3}-0.26X_{1}X_{2}-1.79X_{1}X_{3}-0.14X_{2}X_{3} \end{array}$$

**Figure 1:**
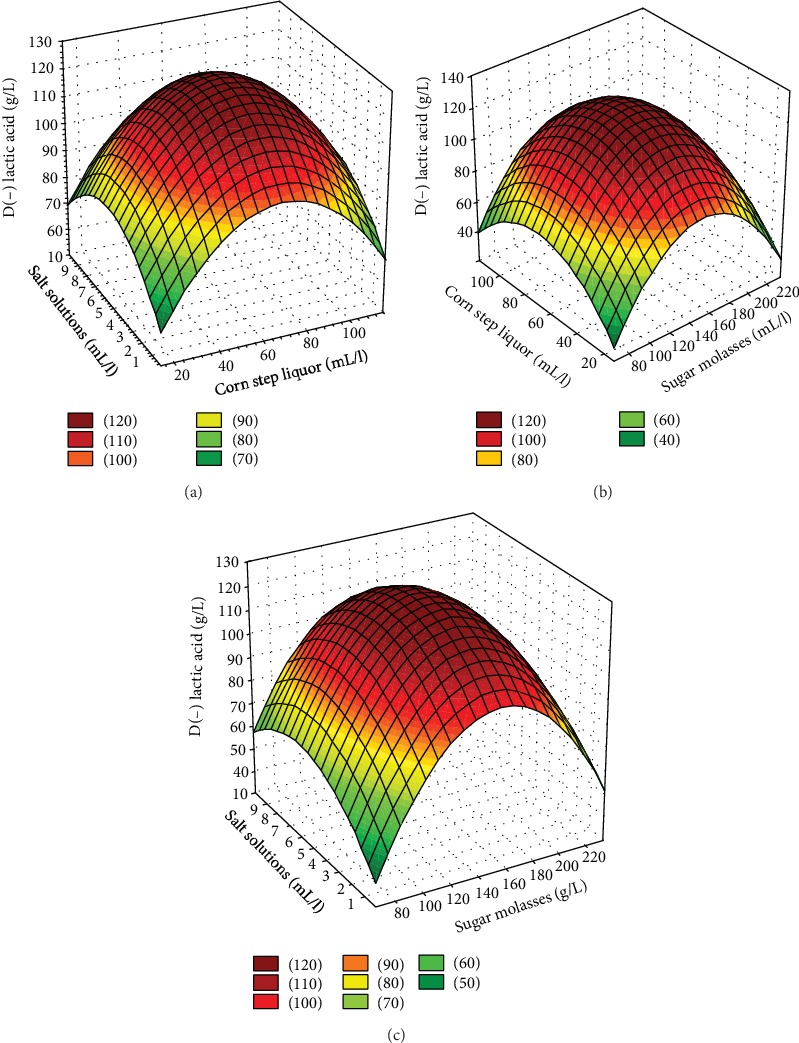
The interaction of media elements on lactic acid production using RSM. (a) Corn steep liquor and salt solution, (b) corn steep liquor and sugar molasses, and (c) salts solution and sugar molasses.

The evaluation of interactions among factors that comprise a process is very important. RSM detects these interactions through three-dimensional response surfaces, which demonstrate the effects of two factors on the response at a given time and assist in determining the degree of parametric interactions on the desired responses [49]. The effects of the independent variables and their interactions on lactic acid production are illustrated in the response surface graphs (Figures 1(a)– 1(c)) constructed from the equation generated according to the coefficient of the linear regression model.

The maximum concentration of lactic acid (120 g/L) was achieved when the initial concentration of sucrose from molasses was approximately 150 g/L, the concentration of corn steep liquor was 65 mL/L, and the concentration of the mineral salt solution was around 5 mL/L. These concentrations correspond to the central point values used in the experimental design. Lactic acid production was reduced when less than 60 g/L of corn steep liquor was provided, as product formation is partially associated with cell growth in this process, in which the nitrogen source plays an important role. This result is in agreement with data described by Lima et al. [50], who applied RSM and achieved maximum D(-) lactic acid production (41.42 g/L) by *Lactobacillus* SMI8 using yeast autolysate and corn steep liquor and found that greater and lower concentrations of the nitrogen source led to a reduction in lactic acid production.

Lactic acid production increased with an increase in the concentration of molasses up to 160 g/L. The reduction in lactic acid production at higher concentrations was likely due to substrate inhibition, which is often reported in batch fermentation processes. Dumbrepatil et al. [30] describe similar results using a mutant *Lactobacillus delbrueckii* to produce lactic acid from molasses, reporting 166 g/L of lactic acid from 190 g/L of molasses and a substantial decrease in lactic acid production when fermentation was carried out with a molasses concentration of 240 g/L.

The mineral salts studied could serve as enzymatic activators, as reported by Lino et al. [21], who found that lactic acid dehydrogenase activity was promoted when adding sodium acetate to the medium for the production of lactic acid by species of *Lactobacillus*.

### 3.3. Effect of Neutralizing Agents on D(-) Lactic Acid Production

**Figure 2:**
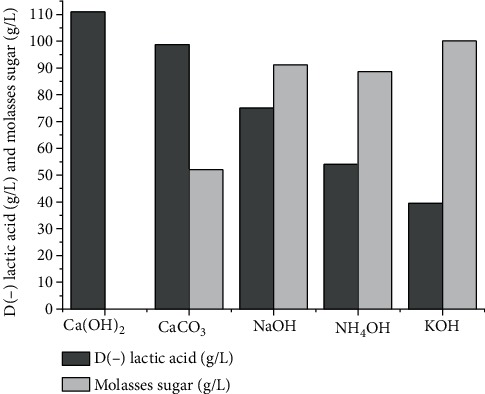
Effect of neutralizing agents on D(-) lactic acid production and residual sugar content by fermentation process with molasses sugar (150 g/L), corn steep liquor (65 mL/L), and salt solution (5 mL/L). Culture conditions: 35°C, 100 rpm, 10% inoculum (*v*/*v*), pH 6.0, and 300 mL of initial work volume.

Figure 2 displays the effects of different neutralizing agents on the batch fermentations.

The sucrose from the molasses was completely consumed when the pH was controlled by Ca(OH)_2_, yielding the highest lactic acid concentration (111.02 g/L). In the presence of CaCO_3_, 98.82 g/L of lactic acid was achieved, but the residual sucrose was substantial (52.00 g/L). When NaOH, NH_4_OH, and KOH were used, the sucrose was not completely converted into lactic acid by *L*. *delbrueckii*, resulting in low yields. These results are in agreement with data reported by Liu et al. [51], who found that Ca(OH)_2_ significantly facilitated D(-) lactic acid production compared to KOH or NH_4_OH using *Escherichia coli*. According to Nakano et al. [52], the pH of the medium is more efficiently neutralized by a divalent cation (Ca^2+^) compared to a monovalent cation (Na^+^, NH_3_^+^).

Some authors consider NaOH-based fermentation to be an environmentally friendly process that avoids generating precipitated waste [9, 53]. However, this agent could increase the osmotic pressure of the medium, causing stress to the microbial cells. Osmotic stress influences cell growth and organic acid production during fermentation, demonstrating an inhibition effect mainly in the late stage of the process [54, 55]. Ca(OH)_2_ and CaCO_3_ are generally applied on the industrial lactic acid fermentation scale, because these neutralizing agents make the downstream process easier and less expensive when compared to other pH-controlling agents. They are also available at a more affordable price than other neutralizing agents [56].

### 3.4. Effect of Temperature and pH on Lactic Acid Fermentation

**Figure 3:**
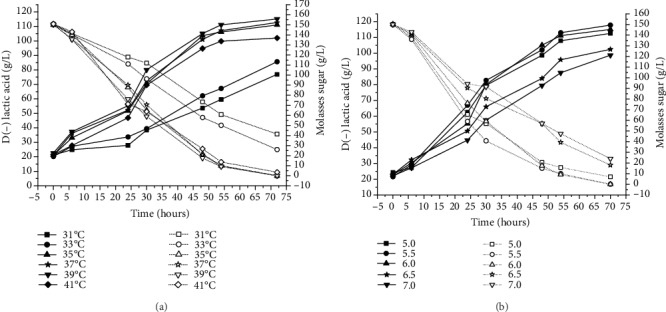
The effects of temperature (a) and pH (b) on lactic acid fermentation. Open symbol: molasses sugar; closed symbol: lactic acid.

Some important factors, such as temperature, pH, agitation, and inoculum size, have to be considered for the efficient production of lactic acid. Temperature and pH are closely related to bacterial growth and consequently affect the production of lactic acid [57]. pH affects the activity of enzymes and the transport of nutrients to microbial cells and also exerts an influence on RNA and protein syntheses [58]. In the present study, fermentations were carried out at 31°C to 41°C, with the optimum temperature for lactic acid production determined to be 39°C, yielding 115.13 g/L. In contrast, higher (41°C) and lower (31 and 33°C) temperatures did not assist in the satisfactory performance of *L*. *delbrueckii* with regard to lactic acid production (Figure 3(a)).

According to results shown in Figure 3(b), the greatest lactic acid production occurred when the pH was maintained at 5.5, reaching a concentration of 117.88 g/L. Higher pH (6.5 and 7.0) had a negative effect on D(-) lactic acid production (Figure 3(b)).

The genus *Lactobacillus* comprises several species for which the optimum temperature for growth is between 35 and 40°C and pH is between 5.5 and 6.0 [59]. Several studies using *Lactobacillus* sp. have demonstrated that the best pH and temperature for lactic acid fermentation are within these ranges. Wee et al. [60] found that the optimum temperature and pH for a batch culture of *Lactobacillus* sp. RKY2 was 6.0 and 36°C, respectively, achieving 153.9 g/L of lactic acid using a medium containing glucose and yeast extract. Tang et al. [10] found that the highest lactic acid yield (0.46 g/g) by *Lactobacillus* sp. was obtained at 37°C and pH 6. Studying lactic acid produced from whey lactose and corn steep liquor by *Lactobacillus* sp. LMI8, Lima et al. [61] found the best temperature and pH to be 39.6°C and 5.9, respectively.

### 3.5. Effects of Agitation and Inoculum Size on Production of D(-) Lactic Acid

**Figure 4:**
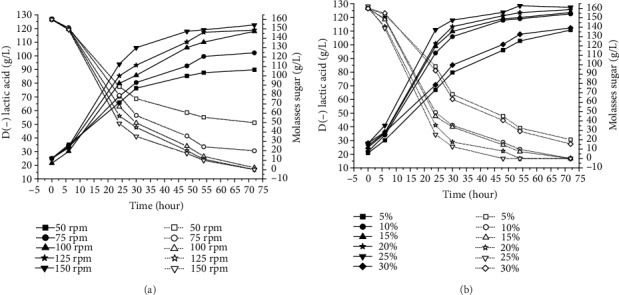
The effects of agitation (a) and inoculum size (b) on lactic acid fermentation. Open symbol: molasses sugar; closed symbol: lactic acid.

Lactic acid production was investigated in some batch fermentation experiments when varying the agitation speed and size of the inoculum, as shown in Figures 4(a) and 4(b).

To determine the effect of inoculum size on lactic acid production, the optimized medium was inoculated with different percentages of culture seed (Figure 4(b)). The smallest (5%) and biggest (30%) inoculum size did not lead to satisfactory lactic acid production. A small inoculum size is reported to result in the low conversion to lactic acid due to inadequate enzymatic efficiency [37, 62]. Panesar et al. [58] report similar findings, with an increase in lactic acid formation and lactose utilization by *Lactobacillus casei* with the increase in inoculum size.

In the present study, the best results were found when the inoculum size was 25% and agitation was 150 rpm, achieving 122.55 g/L and 127.35 g/L of D(-) lactic acid, respectively. As the medium was composed of waste products with particles that tend to precipitate, more vigorous agitation promoted greater homogenization, thereby increasing contact between the cells and components of the medium. A lower agitation speed could result in an insufficient homogenization and the substrate would not be completely utilized, as demonstrated when 50 rpm and 75 rpm were used. Indeed, no residual sugar was observed at the end of the process when the highest agitation levels were used. Agitation is also important for mass and heat transfer [63].

### 3.6. Study of Fed-Batch Fermentation Strategies

The multipulse fed-batch strategy was performed with a low initial molasses concentration, and applying two pulses when the sucrose content was almost or completely exhausted. With this method, the highest concentration of D(-) lactic acid (161.93 g/L) was achieved after 48 hours of fermentation. However, production of 136.15 g/L of lactic acid was found at 30 hours, with the best productivity (4.54 g/L·h). When combined with the control of pH by Ca(OH)_2_, this method avoids excessive osmotic pressure that medium components could cause in the bacterial cells. Therefore, the phenomenon of substrate inhibition was avoided by providing small amounts of sucrose from molasses in the log phase of *L*. *delbrueckii* growth. The fed-batch strategy promotes a 33.73% increase in lactic acid production compared to a batch culture. Many studies report improvements in fermentation performance employing fed-batch strategies. Using a mutant *Bacillus* strain grown in a glucose and peanut meal medium, Assavasirijinda et al. ([9] performed multipulse fed-batch fermentation and found productivity of approximately 3.02 g/L·h at 16 h, reaching a final D(-) lactic acid concentration of 142.05 g/L. Using the twice fed-batch feeding strategy for *Sporolactobacillus laevolacticus* DSM442, Li et al. [64] achieved 144.4 g/L of D(-) lactic at 35 hours of fermentation as well as productivity of 4.13 g/L·h.  Table 5 displays the best result of each fed-batch strategy in the present study, which was achieved at different fermentation times.

**Figure 5:**
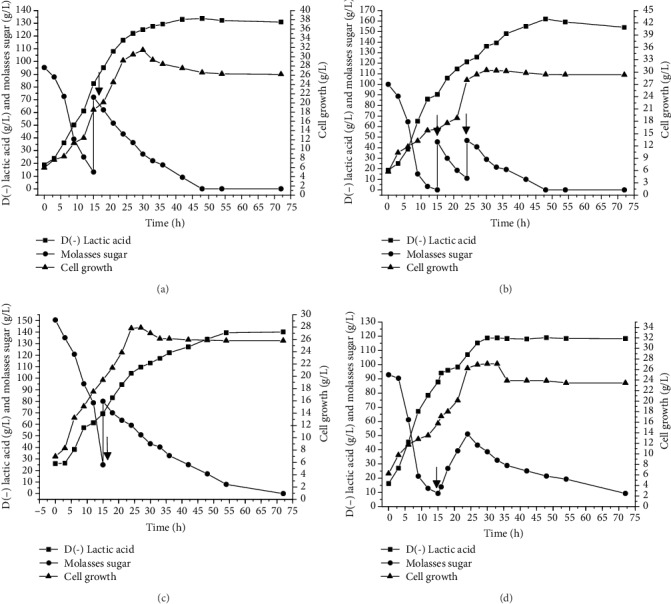
Fed-batch fermentations of D(-) lactic acid by *L. delbrueckii*. (a, c) Pulse. (b) Multipulse. (d) Continuous feed rate. Culture conditions: 39°C, 150 rpm, 25% inoculum (*v*/*v*), 300 mL of initial work volume, and pH 5.5 controlled by Ca(OH)_2_. The arrows represent when the pulse occurred (a–c) or the beginning of the fed-batch phase (d).

It is evident that a high substrate concentration at the beginning of the process is not advantageous [22, 65]. In the present study, the single-pulse strategy that was initiated with a higher initial substrate concentration (pulse fed-batch II) took more time to reach the same concentration of lactic acid compared to the pulse fed-batch with a low initial substrate concentration (fed-batch I). Likewise, Ye et al. [65] found an improvement in productivity and production (3.2 g/L h and 140.4 g/L, respectively) using a single-pulse fed-batch strategy compared to a batch culture (2.6 g/L h and 118.2 g/l, respectively) involving *Bacillus coagulans* C106.  Figure 5 displays the fed-batch fermentation kinetics, demonstrating D(-) lactic acid production, sucrose consumption, and cell growth rate.

The constant feed rate fed-batch was not satisfactory, yielding the lowest D(-) lactic acid production among all fed-batch strategies. When the feed supply was stopped, the microbial cells were entering the stationary phase, declining thereafter. This result is in agreement with data described by Ding and Tan [19], who also did not achieve good results when employing this strategy on *Lactobacillus casei* fermentation.

The final culture medium (from multipulse fed-batch fermentation) was analyzed and high optical purity of the D(-) lactic acid was found (97.66% of D(-) and 2.34% of L(+) lactic acid).

## 4. Conclusion

The present findings demonstrate an efficient and low-cost process for the production of high optical purity D(-) lactic acid by *L. delbrueckii,* which was able to grow and convert sucrose from waste products (molasses and corn steep liquor) into D(-) lactic acid. This is a very economical process, since no pretreatment of the waste products is required. High levels of lactic acid and high productivity were achieved. Moreover, the pH neutralizer employed [Ca(OH)_2_] has the advantage of being available at a very affordable cost and contributes to maintaining low osmotic pressure in the culture medium, which is very important in avoiding stress in microbial cells. The multipulse fed-batch strategy conducted with the optimum conditions studied is an easy, effective method to improve D(-) lactic acid production.

## Figures and Tables

**Table 1:** Culture conditions on the study of the parameters' effect on lactic acid production.

Parameter	*T* (°C)	pH	Agitation (rpm)	Inoculum (%)	Change rate
Temperature range	31-41	6.0	100	10	2°C
pH range	39	5.0-7.0	100	10	0.5 units
Agitation range	39	5.5	50-150	10	25 rpm
Inoculum size range	39	5.5	150	5-30	5%

**Table 2:** Effects of carbon and nitrogen sources on D(-) lactic acid production, productivity, and yield by *L. delbrueckii.*

Carbon sources	Carbohydrates	D(-) lactic acid (g/L)	Productivity (g/L·h)	Yield *Y*_p/*s*_ (g/g)	Residual sugar (g/L)
Sugar molasses	46.9 (g/100 mL)^a^	112.84 ± 3.07	2.35 ± 0.06	0.88 ± 0.01	12.12 ± 1.22
Commercial sucrose	100 (g/100 g)^a^	111.37 ± 1.15	2.32 ± 0.02	0.96 ± 0.02	24.98 ± 1.22
Whey	76.59 (g/100 g)^b^	100.08 ± 2.06	2.08 ± 0.04	0.89 ± 0.02	28.32 ± 0.51
Nitrogen sources	Percentage of *N*				
Yeast autolysate	7.2%	102.79 ± 1.92	2.14 ± 0.04	0.87 ± 0.00	22.08 ± 1.88
Corn steep liquor	4.69%	101.06 ± 3.36	2.10 ± 0.07	0.86 ± 0.05	23.41 ± 1.85
Proflo	11.15%	75.74 ± 1.20	1.57 ± 0.03	0.75 ± 0.04	39.11 ± 4.04
Peanut flour	7.63%	66.03 ± 0.47	1.37 ± 0.01	0.68 ± 0.03	44.11 ± 1.84
Soybean flour	6.40%	62.65 ± 1.38	1.30 ± 0.03	0.71 ± 0.07	52.43 ± 3.47
Ammonium nitrate	34%	52.60 ± 3.40	1.09 ± 0.07	0.57 ± 0.13	48.92 ± 2.36
Urea	46.62%	49.16 ± 1.30	1.02 ± 0.03	0.66 ± 0.04	66.56 ± 1.07
Ammonium sulfate	21%	37.09 ± 0.10	0.77 ± 0.00	0.56 ± 0.03	74.46 ± 1.23

Culture conditions: GYP medium with 140 g/L of carbon source and 15.25% of nitrogen contained on each nitrogen sources, 5% of CaCO_3_, 50 mL of work volume, at 35°C, and stationary fermentation with an initial pH of 7.0 for 48 hours. Types of carbohydrate: ^a^sucrose and ^b^lactose.

**Table 3:** RSM design matrix with real/coded values and experimental results.

Run	Dependent variables			D(-) lactic acid	Productivity	*Y*_*p*/*s*_	Residual sucrose
	*X*_1_	*X*_2_	*X*_3_	g/L	g/L·h	g/g	g/L
1	100.00 (-1)	35.00 (-1)	2.20 (-1)	82.30	1.71	0.86	21.66
2	200.0/0 (1)	35.00 (-1)	2.20 (-1)	82.61	1.72	0.73	107.22
3	100.00 (-1)	95.00 (1)	2.20 (-1)	79.53	1.66	0.95	32.40
4	200.00 (1)	95.00 (1)	2.20 (-1)	82.99	1.73	0.98	130.36
5	100.00 (-1)	35.00 (-1)	7.80 (1)	88.00	1.83	0.98	25.44
6	200.00 (1)	35.00 (-1)	7.80 (1)	85.35	1.78	0.91	122.48
7	100.00 (-1)	95.00 (1)	7.80 (1)	88.85	1.85	0.94	21.26
8	200.00 (1)	95.00 (1)	7.80 (1)	80.93	1.69	0.93	128.96
9	150.00 (0)	65.00 (0)	0.13 (-1.68)	102.03	2.13	0.97	60.27
10	150.00 (0)	65.00 (0)	9.87 (+1.68)	110.21	2.30	0.99	53.55
11	150.00 (0)	14.60 (-1.68)	5.00 (0)	82.39	1.72	0.97	80.36
12	150.00 (0)	115.40 (+1.68)	5.00 (0)	102.77	2.14	0.98	60.77
13	66.00 (-1.68)	65.00 (0)	5.00 (0)	68.88	1.44	0.97	10.61
14	234.00 (+1.68)	65.00 (0)	5.00 (0)	75.02	1.56	0.49	111.38
15	150.00 (0)	65.00 (0)	5.00 (0)	124.56	2.60	0.96	36.30
16	150.00 (0)	65.00 (0)	5.00 (0)	118.75	2.47	0.94	39.28
17	150.00 (0)	65.00 (0)	5.00 (0)	122.56	2.55	0.96	37.79

*X*_1_ = sugar molasses (g/L of sucrose), *X*_2_ = corn steep liquor (mL/L), *X*_3_ = salt solution (mL/L), and *Y*_*p*/*s*_ = yield. Culture conditions: initial pH at 7.0, 5% of CaCO_3_ used as pH neutralizer, 50 mL of work volume, 10% inoculum (*v*/*v*), and 35°C. Fermentation was carried under stationary condition for 48 hours. Coded values were present in parenthesis.

**Table 4:** ANOVA for the regression model with estimated regression coefficients for D(-) lactic acid production.

D(-) lactic acid production					
Terms	Sum of squares	Degree of freedom	Media of squares	*F* calc	*p*
Model	4505.71	9	500.63	13.08	0.001
Error	267.86	7	38.26	-	-
Lack of fit	250.44	5	50.08	5.74	0.154
Pure error	17.42	2	8.71	-	-
Total	4773.57	16	-	-	-
*R*2 = 0.94					
Terms		Coefficient			*p* value
Intercept		122.24			0.0000^∗^
*X*_1_		0.25			0.8817
*X*_1_ *X*_1_		-18.67			0.0000^∗^
*X*_2_		2.07			0.2554
*X*_2_ *X*_2_		-11.37			0.0004^∗^
*X*_3_		2.15			0.2385
*X*_3_ *X*_3_		-6.59			0.0090^∗^
*X*_1_ *X*_2_		-0.26			0.9069
*X*_1_ *X*_3_		-1.79			0.4394
*X*_2_ *X*_3_		-0.14			0.9481

*X*_1_ = sugar molasses (g/L of sucrose), *X*_2_ = corn steep liquor (mL/L), *X*_3_ = salt solution (mL/L). ^∗^Statistically significant at 95% of probability level.

**Table 5:** Fed-batch fermentation strategies on D(-) lactic acid production.

	Batch culture	Pulse fed-batch I	Pulse fed-batch II	Multipulse fed-batch	Constant feed rate fed-batch
Fermentation time (h)	33	48	54	48	30
D(-) lactic acid (g/L)	121.08	138.84	139.42	161.93	118.79
Productivity (g/L·h)	3.36	2.89	2.58	3.37	3.96
Yield *Y*_p/s_ (g/g)	0.61	0.78	0.55	0.81	0.61
Residual sucrose (g/L)	0	0	7.97	0	31.63
Cell growth (g/L)	22.55	26.57	25.80	29.42	27.07
Final broth volume (ml)	400	520	480	580	550

Pulse fed-batch I started with 95 g/L of initial molasses sugar; pulse fed-batch II started with 150 g/L of initial molasses sugar.

## Data Availability

All data in the manuscript are available.
